# A Novel Donor-Acceptor Thiophene-Containing Oligomer Comprising Dibenzothiophene-*S,S*-dioxide Units for Solution-Processable Organic Field Effect Transistor

**DOI:** 10.3390/molecules27092938

**Published:** 2022-05-04

**Authors:** Xia Luo, Zongfan Duan, Kang Li, Gang He, Zhenzhen Liu, Hong Luo, Jingyu Zhang, Jiani Liang, Qian Guo, Jing Liu, Kai Ding

**Affiliations:** 1 School of Materials Science and Engineering, Xi’an University of Technology, Xi’an 710048, China; luoxia1223@163.com (X.L.); 18710493483@163.com (K.L.); version521@live.com (G.H.); ycfszd2327@163.com (Z.L.); 15198381707@139.com (H.L.); ginny1997@163.com (J.L.); gq1213440601@163.com (Q.G.); liujyu2022@163.com (J.L.); kai13468697372@sina.com (K.D.); 2 Materials Corrosion and Protection Key Laboratory of Sichuan Province, Zigong 643000, China; zjy312@suse.edu.cn

**Keywords:** thiophene, oligomer, synthesis, semiconductor material, organic field effect transistor

## Abstract

A π-conjugated thiophene-containing oligomer with a D-A-D-A-D (D: donor, A: acceptor) architecture, namely, 2,6-bis{[*4-(7-n-hexylthiophen-2-yl)thiophen-2-yl*]-(dibenzothiophene-5,5-dioxide-3,3΄-diyl)}-bis((2-ethyl-hexyl)oxy)benzo[*1,2-b:4,5-b’*]dithiophen (BDT(DBTOTTH)_2_), was synthesized by Stille coupling reactions. There are obvious shifts in the Ultraviolet-visible (UV-vis) and photoluminescence (PL) spectra of the thin film relative to its solution, indicating the existence of the π-π stacking in the solid state of the oligomer BDT(DBTOTTH)_2_. The optical band gap of the oligomer determined from its absorption onset in UV-Vis spectra is 2.25 eV. It agrees with the value of 2.29 eV determined from the cyclic voltammetry (CV) measurement. Its highest occupied and lowest unoccupied molecular orbital (HOMO/LUMO) energy levels, which were calculated from its onset of oxidation and reduction waves in CV curve, are −5.51 and −3.22 eV, respectively. The oligomer is a P-type semiconductor material with a good thermal stability and solubility, which can be used to fabricate organic field effect transistors (OFETs) by the spin coating technique. The OFET with *n*-octadecanylltrichlorosilane (OTS)-modified SiO_2_ dielectric layer exhibited a mobility of 1.6 × 10^−3^ cm^2^/Vs.

## 1. Introduction

Over the past decade, π-conjugated polymers and oligomers have been extensively investigated because of their unique photo-electronic properties and potential applications in different organic electronic devices, such as organic light emitting diodes (OLEDs), solar cells and organic field effect transistors (OFETs) [1,2,3]. The previous work on the design of p-type organic semiconductor molecules suggests that the incorporation of an electron-accepting unit into the molecular backbone of a π-conjugated polymer/oligomer with electron-donating property can result in a small lowering of its highest occupied molecular orbital (HOMO) level, and improve its stability against oxidation. More significantly, it has been confirmed that the formation of donor-acceptor (D-A) structure is beneficial to promote the charge transfer between the donor unit and the acceptor unit in molecules, and obtain a good charge mobility [4]. Therefore, many electron-accepting units including benzothiadiazole [5,6], thiadiazolopyridine [7], naphthalene bisimide [8], thieno-thiadiazole [9], diketopyrrolopyrrole [10,11,12], bithiophenesulfonamide [13] dithiazole [14], etc., have been introduced into the frameworks of π-conjugated polymers/oligomers. The obtained compounds are used as the excellent organic semiconductor materials in a variety of electronic devices. Among various organic semiconductor materials, π-conjugated thiophene-containing oligomers have been extensively explored as an active organic semiconductor material due to their easiness in preparation, purification, chemical structural modification, and moderation of energy levels and optical/electronic properties [15,16,17,18]. In this case, it is an interesting subject research on design, synthesis and properties of thiophene-containing oligomers with D-A structure.

Thiophene-*S*,*S*-dioxides have been examined as an electron-accepting unit. The oligomers containing thiophene-*S*,*S*-dioxide moieties have the smaller energy gaps, the higher electron affinities and the greater stability than their precursor oligothiophenes [19,20,21]. The oxidation of the sulfur atom of dibenzothiophene with coplanar rings can yield dibenzothiophene-*S*,*S*-dioxide (DBTSO), which is a novel electron-accepting unite. Recently, the DBTSO acceptor unit as a central core has been incorporated into the backbone of electron donor oligomers such as fluorene [22,23,24,25], carbazole [26], arylamine [27], quinoxaline or pyrazine [28] via co-oligomerization to afford highly efficient OLED materials. Furthermore, our group previously synthesized a D-A-D type oligomer with a DBTSO core and two end-caped phenylthiophene substitutes. Although the optical energy band gap of this oligomer is relative wide (2.52 eV), its bulk-heterojunction (BHJ) solar cell can provide a relative high power conversion efficiency of 0.84% [29,30]. Overall, DBTSO is an ideal electron-accepting unite, and can be used to design and build some potential semiconductor materials with a D-A structure. However, to our knowledge, little attention has been paid to the design and synthesis of thiophene-containing oligomers with more than two DBTSO electron-accepting unites. In addition, there are also no reports on their application in OFET devices.

**Figure 1:**
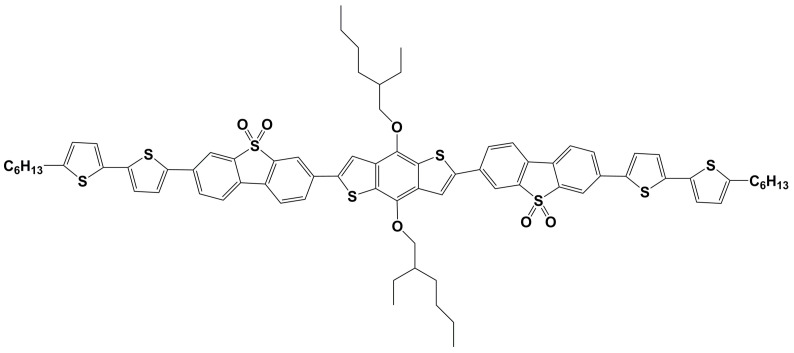
Molecular structure of oligomer BDT(DBTOTTH)_2_.

In this work, a novel π-conjugated thiophene-containing oligomer with two DBTSO electron-accepting unites and a D-A-D-A-D architecture (Figure 1) has been synthesized. It is 2,6-bis{[*4-(7-n-hexylthiophen-2-yl)thiophen-2-yl*]-(dibenzothiophene-5,5-dioxide-3,3′-diyl)}-bis((2-ethyl-hexyl)oxy)benzo[*1,2-b:4,5-b′*]dithiophen (BDT(DBTOTTH)_2_), which has a benzo[*1,2-b:4,5-b′*]dithiophene donor core, two DBTSO acceptor intermediaries and two end-capped hexyl dithienyl donor units. Its photophysical properties, energy band gap, molecular orbital energy levels and thermal stability were then characterized using Ultraviolet-visible (UV-vis), photoluminescence (PL) spectra, cyclic voltammetry (CV) and thermogravimetric analysis (TGA). Considering that the oligomer has a large linear π-conjugated and D-A-D-A-D structure, and a good solubility deriving from of hexyl and 2-ethyl-hexyloxy groups, it would be a solution-processable semiconductor material. Therefore, the oligomer BDT(DBTOTTH)_2_ was further used to fabricate an OFET device by a simple spin coating technique, and a mobility of 1.6 × 10^−3^ cm^2^/Vs was obtained.

## 2. Experimental

### 2.1. Materials and Characterization

All chemicals were purchased from Sigma-Aldrich without any further purification. Tetrahydrofuran (THF) and toluene were freshly distilled from sodium and benzophenone, *N*,*N*,-dimethylformamide (DMF) and dichloromethane were distilled from CaH_2_ under N_2_ atmosphere prior to use. Thin-layer chromatography (TLC) was performed using pre-coated silica gel plates (Merck Kieselgel 60F_254_) and column chromatography was employed using silica gel (Merk, 130–270 mesh). Nuclear magnetic resonance (NMR) spectra for ^1^H and ^13^C spectra were recorded on spectrometer ((INOVA-400MHz)) using deuterated chloroform (CDCl_3_) as a solvent. Additionally, mass spectra were acquired on a matrix-assisted laser desorption/ionization-time-of-flight (Bruker ultraflextreme MALDI-TOF) mass spectrometer. All chemical shifts were shown in parts per million (ppm) with tetramethylsilane as an internal standard. The UV-vis spectra of the oligomer in a chloroform solution (10^−6^ M) and a thin film were recorded on a U-3900H spectrophotometer. The thickness of the film is about 50 nm, which drop-casted from the tetrachlorethan solution on a quartz substrate. The PL spectra of the oligomer in the solution and thin film were then measured with a FLUOROMAX-4 fluorospectrophotometer (λ_excitation_ = 318 nm). The CV measurement of the oligomer was performed on PARSTAT-4000A using a three-electrode cell at room temperature. In CV measurement, the oligomer was dissolved in an anhydrous dichloromethane in a concentration of 10^−3^ M, containing 0.1 M of tetrabutylammonium hexafluorophosphate (Bu_4_NPF_6_) as the supporting electrolyte. The working electrode and counter electrode was a platinum stick and platinum wire, respectively. In addition, a calomel electrode was used as the reference electrode, which had been calibrated against ferrocene/ferrocenium. The TGA was performed using a SHIMADZU, DTG-60H instrument at a heating rate of 10 °C/s in nitrogen atmosphere. Transistor measurements were performed in air using an Aglient 4155C semiconductor parameter analyzer.

### 2.2. Oligomer Synthesis

**Figure 2:**
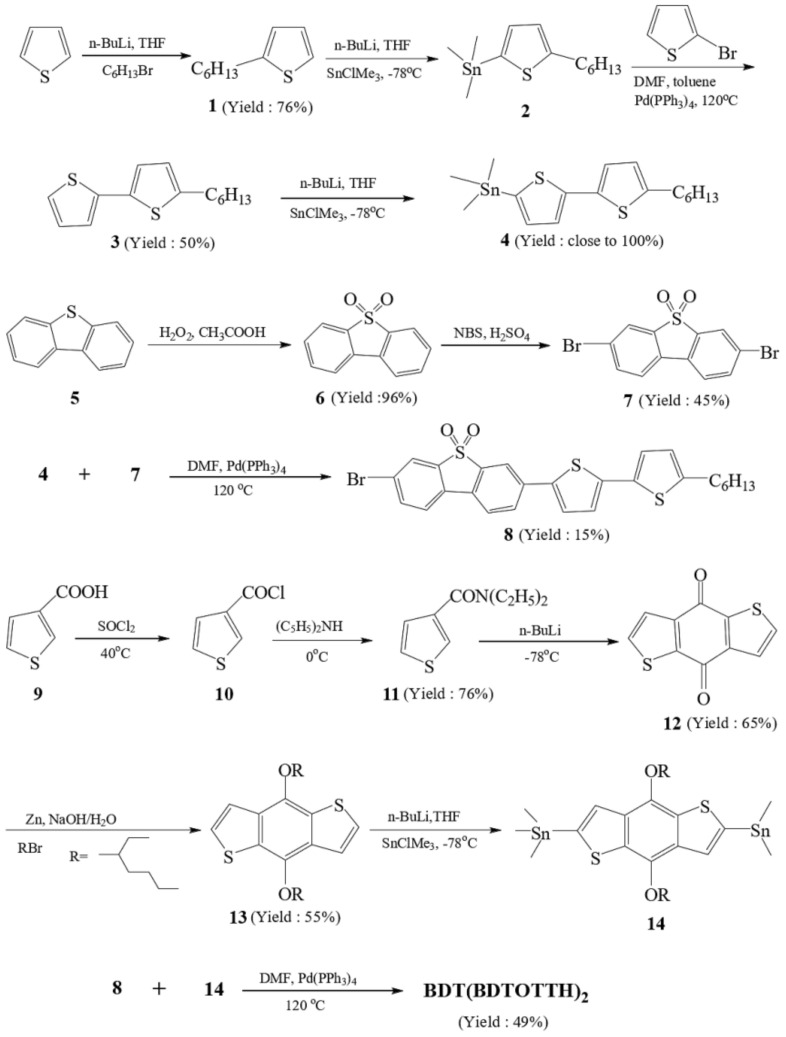
Synthetic pathway for the oligomer BDT(DBTOTTH)_2_.

Figure 2 outlines the synthetic pathway of the oligomer BDT(DBTOTTH)_2_. 2-Hexylthiophene (**1**), 2-(trimethylstannyl)-5-hexylthiophene (**2**) and 5-hexyl-2,2′-bithiophen (3) were prepared according to literature procedures [31]. The compound (**3**) reacted with *n*-butyllithium and trimethyltin chloride to produce 5-(trimethylstannyl)-5′-hexyl-2,2′-bithiophene (**4**) [32]. Because the stannyl compound (**4**) is liable to acids, it was used without any further purification. 3,7-Dibromodibenzothiophene-*S*,*S*-dioxide (**7**) was obtained by two steps: the oxidation of dibenzothiophene ((**5**)→(**6**)) and bromination with bromine/acetic acid((**6**)→(**7**)) [33]. The intermediate, 3-bromo-7-(5′-hexyl-2,2′-bithiophen-5-yl)dibenzo[*b,d*]thiophene *S,S*-dioxide (**8**), was prepared by the Stille cross-coupling reaction of 5-trimethylstannyl-5′-hexyl-2,2′-bithiophene (**4**) with 3,7-dibromodibenzothiophene-*S*,*S*-dioxide (**7**) in the presence of Pd(PPh_3_)_4_ catalyst. The 3-thiophene carbonyl chloride (**10**) was obtained from the reaction of commercially 3-thiophenecarboxylic acid (**9**) with thionyl chloride. The amidation reaction of 3-thiophene carbonyl chloride (**10**) with diethylamide gave the corresponding *N*,*N*-diethylthiophene-3-carboxamide (**11**). Benzo[*2,3-b:5,6-b*′]dithiophene-4,8-dione (**12**) was prepared by the reaction of *N*,*N*-diethylthiophene-3-carboxamide (**11**) with *n*-butyllithium at −78 °C under nitrogen atmosphere. The soluble 4,8-bis(octyloxy)benzo[*1,2-b:4,5-b*′]dithiophene (**13**) was obtained in moderate yield from benzo[*2,3-b:5,6-b*′] dithiophene-4,8-dione (**12**) by being treated with a mixture of Zn powder, NaOH and deionized water, and finally reacted with 2-ethylhexyl bromide in the presence of (*n*-Bu)_4_NBr catalyst [34]. The compound 2,6-bis(trimethylstannane)-4,8-bis((2-ethylhexyl)oxy)benzo[*1,2-b:4,5-b*′]dithiophene (**14**) was prepared using a similar methodology to stannane (**2**) [35]. The synthesis of the oligomer BDT(DBTOTTH)_2_ was also commenced using the Stille cross-coupling catalyzed by Pd(PPh_3_)_4_. To ensure completion of the coupling reaction, an excess of the bromide (**8**) was used [36].

#### 2.2.1. Synthesis of 3-bromo-7-(5′-hexyl-2,2′-bithiophen-5-yl)dibenzo[b,d]thiophene-*S,S*-dioxide (**8**)

5-Trimethylstannyl-5′-hexyl-2,2′-bithiophene (2.19 g, 5.33 mmol), 3,7-dibromodibenzothiophene-*S*,*S*-dioxide (5.98 g, 16 mmol), Pd(PPh_3_)_4_ (46 mg, 0.04 mmol) and anhydrous *N*,*N*-dimethylformamide (DMF, 50 mL) were successively added into a 100 mL three-neck round-bottomed flask. After deoxygenating with dry nitrogen for 30 min, the mixture was stirred at 120 °C for 30 h under nitrogen atmosphere. The reactants were cooled to room temperature and the resulting yellow solid was then collected by filtration. The filtrate was extracted with dichloromethane (100 mL) for 3 times. The organic layer was dried over MgSO_4_, filtered and evaporated. Two parts of solid were combined and then purified by column chromatography using petroleum ether/dichloromethane (3:1) as eluent (Rf = 0.52). A yellow solid of 0.43 g was obtained with a yield of 15%. ^1^H NMR (CDCl_3_, 400 Hz, δ/ppm): 8.28 (s, 1H), 8.15 (s, 1H), 8.03 (d, *J* = 6.0 Hz, 1H), 7.94 (d, *J* = 6.4 Hz, 1H), 7.79 (d, *J* = 6.4 Hz, 1H), 7.74 (d, *J* = 6.4 Hz, 1H), 7.65 (d, *J* = 7.6 Hz, 1H), 7.62 (d, *J* = 7.6 Hz, 1H), 7.51 (d, *J* = 7.6 Hz, 1H), 7.46 (d, *J* = 7.6 Hz, 1H), 2.50 (t, *J* = 8.4 Hz, 2H), 1.58–1.81 (m, 2H), 1.17–1.45(m, 6H), 0.72–0.94 (m, 3H). ^13^C NMR (CDCl_3_, 100 MHz, ppm): *δ* 145.21, 144.28, 142.90, 140.87, 139.25, 132.45, 129.85, 124.03, 123.48, 122.32, 120.35, 117.91, 115.88, 114.03, 40.68, 31.90, 29.67, 22.67, 14.09. MALDI-TOF MS (*m*/*z*): calcd for C_26_H_23_BrO_2_S_3_, 542.0044, found; 542.0042. Melting point: 370–372 °C.

#### 2.2.2. Synthesis of the Oligomer BDT(DBTOTTH)_2_

2,6-Bis(trimethylstannane)-4,8-bis((2-ethylhexyl)oxy)benzo[*1,2-b:4,5-b*′]dithiophene (0.43 g, 0.56 mmol), 3-bromo-7-(5′-hexyl-2,2′-bithiophen-5-yl)dibenzo[*b,d*]thiophene-*S,S*-dioxide (0.82 g, 1.5 mmol), anhydrous DMF (50 mL) and Pd(PPh_3_)_4_ (17 mg, 0.015 mmol) were added to a 100 mL three-neck round-bottomed flask in turn. The mixture was deoxygenated with nitrogen for 30 min, and then stirred at 120 °C for 30 h under nitrogen. The resulting solid was collected by filtration, and washed with KF solution (100 mL, 8%), water (100 mL) and dichloromethane (50 mL) in turn. The obtained crude product was then purified by column chromatography using petroleum ether/dichloromethane (1:1) as an eluent for 3 times (Rf = 0.43). A yellowish-brown solid of 0.38 g was obtained with a yield of 49%. The melting point (m.p.) is higher than 270 °C. ^1^H NMR (CDCl_3_, 400 Hz, δ/ppm): 8.33 (s, 4H), 7.87 (d, *J* = 5.6 Hz, 4H), 7.80 (d, *J* =6.4 Hz, 4H), 7.70 (s, 2H), 7.63 (d, *J* =7.2 Hz, 2H), 7.58 (d, *J* = 7.2 Hz, 2H), 7.48 (d, *J* =7.2 Hz, 2H), 7.42 (d, *J* = 7.2 Hz, 2H), 4.17–4.22(m, 2H), 4.05–4.14 (m, 2H), 2.43 (t, *J* = 8.4 Hz, 4H), 1.84–2.15 (m, 2H), 1.54~1.64 (m, 8H), 1.04~1.37 (m, 24H), 0.64~0.89 (m, 18H). ^13^C NMR (CDCl_3_, 100 MHz, ppm): *δ* 144.41, 143.25, 142.51, 137.33, 135.03, 135.00, 134.32, 132.72, 131.12, 129.05, 128.84, 126.89, 125.78, 124.88, 123.11, 122.85, 119.71, 119.04, 72.01, 39.30, 38.99, 32.14, 31.74, 30.79, 29.91, 27.94, 22.93, 19.40, 14.09. MALDI-TOF MS (*m*/*z*): calcd for C_78_H_82_O_6_S_8_, 1370.3877; found, 1370.3874.

#### 2.2.3. Fabrication of OFET Device

**Figure 3:**
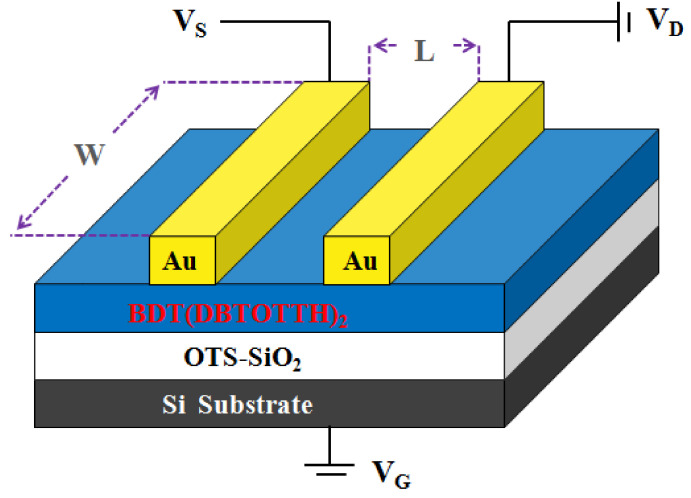
Schematic configuration of the top contact OFET device based on BDT(DBTOTTH)_2_.

The cost of device fabrication is a vital factor for the application of organic electron devices. The spin-coating has been proven to be an easier and low cost fabrication process compared with the vacuum evaporation. In this work, the oligomer BDT(DBTOTTH)_2_ was used to fabricate OFET devices by the spin coating method. As shown in Figure 3, the OTFT device has ‘‘top contact’’ configuration. The *n*-doped silicon substrate functions as the gate electrode, and a thermally grown 300 nm silicon dioxide (SiO_2_) modified by an *n*-octadecanylltrichlorosilane self-assembled monolayer layer (OTS-SiO_2_) works as the insulating dielectric layer. The OTS-SiO_2_/Si was prepared by SiO_2_/Si being treated with a toluene solution of OTS (10 mg/mL) at 60 °C for 20 min. Its capacitance per unit area is 11.0 nF/cm^2^. The thin film of BDT(DBTOTTH)_2_ was fabricated on OTS-SiO_2_/Si substrate by spin coating in a tetrachlorethan solution (2 mg/mL) at 1500 rpm for 100 s and then annealed at 150 °C for 30 min. As a result, a BDT(DBTOTTH)_2_ semiconductor active layer with a thickness of about 80 nm was obtained. The source (S) and drain (D) Au electrodes were deposited onto BDT(DBTOTTH)_2_ layer through a shadow mask by vacuum thermal deposition method. The thickness of Au electrode is about 100 nm, and the channel length and width of the OTFT device are 50 and 500 μm, respectively.

### 2.3. Results and Discussion

#### 2.3.1. Synthetic Methodology

For the synthesis of thiophene-containing oligomers, one of the most useful procedures in the formation of C-C σ-bonds is the metal-promoted coupling reaction of organic halides. In this work, the palladium(0)-catalyzed Stille cross-coupling reaction was used as a main reaction type to prepare the oligomer BDT(DBTOTTH)_2_ due to its tolerance to a variety of functional groups (e.g., CO_2_R, CHO, OH, SO_2_). In order to obtain a single coupling product (**8**) as much as possible, an excess of the bromide (**7**) was used. The optimum mole ratio of stannyl compound (**5**) and bromide (**7**) was 1:3. The compound (**8**) and the target oligomer BDT(DBTOTTH)_2_ were soluble in common solvents such as dichloromethane, chloroform, THF and tetrachlorethan. They were purified by column chromatography, owing to the contribution from *n*-hexyl or/and 2-ethyl-hexyloxy groups.

#### 2.3.2. Photophysical Properties

**Figure 4:**
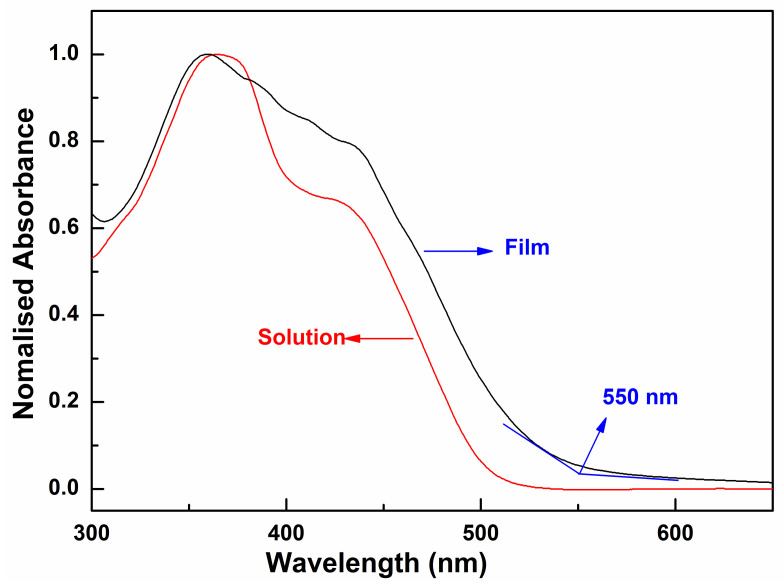
UV-vis absorption spectra of oligomer BDT(DBTOTTH)_2_.

**Figure 5:**
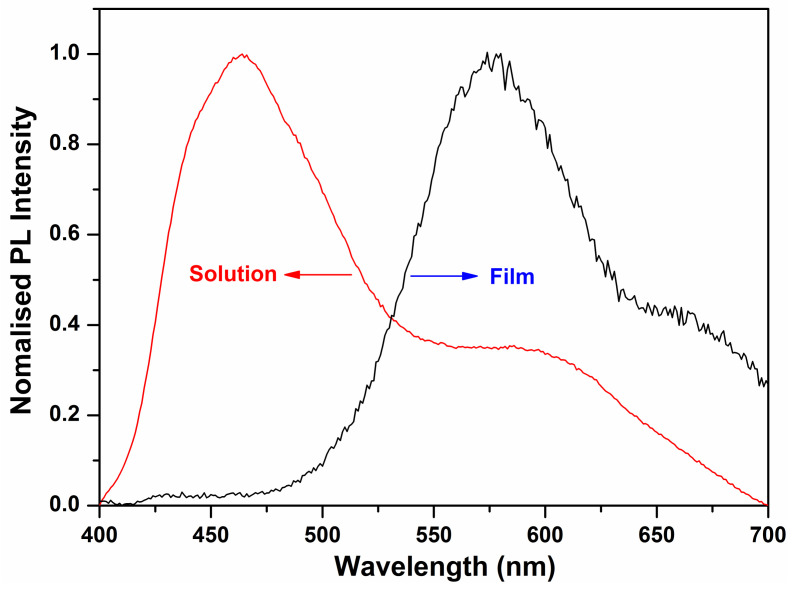
PL spectra of the oligomer BDT(DBTOTTH)_2_.

As shown in Figure 4, the oligomer in dilute CH_2_Cl_2_ solution has the absorption maximum value at 359 nm and a broad shoulder peak at 425 nm, whereas in the thin film, the absorption band of the oligomer becomes relatively broader and less structured. The maximum absorption peak displays a blue-shift about 7 nm relative to that of its corresponding solution. The blue-shift should attribute to the formation of the H-aggregate in the solid state [37], which is usually observed in excellent semiconductor materials. In H-aggregates, since the molecules are closely π-stacked in a face-to-face alignment, the neighboring molecules also interact in the ground state [38]. The absorption onset (λ_edge_) of the oligomer film in UV-Vis spectra is 550 nm. According to Equation (1),

(1) $$\;E_{g}^{opt}\left(\mathrm{eV}\right)=1240/\lambda_{\mathrm{edge}}\left(\mathrm{nm}\right)$$

the optical band gap of the oligomer was calculated to be 2.25 eV. This band gap is moderate, and very close to that of pentacene (2.2 eV), which is the most well-known OFET material [39]. The pronounced changes in the absorption spectra are a result of the delocalization of the exciton within co-facial stacks induced by the π-π interactions, which is also evidenced by a related red-shift of the PL spectra (Figure 5). The emission spectrum of the oligomer BDT(DBTOTTH)_2_ shows strong blue-green fluorescence in the diluted solution, and exhibits a maximum emission peak at 464 nm with a broad shoulder peak ranging from 540 to 610 nm. The emission spectrum of the oligomer BDT(DBTOTTH)_2_ in the thin film has a similar pattern as that in solution except for an obvious red shift. The maximum emission peak of the oligomer in the film is located at 578 nm. Compared with that in the solution, the emission spectrum is bathochromically shifted by 114 nm.

#### 2.3.3. Electrochemical Properties

**Figure 6:**
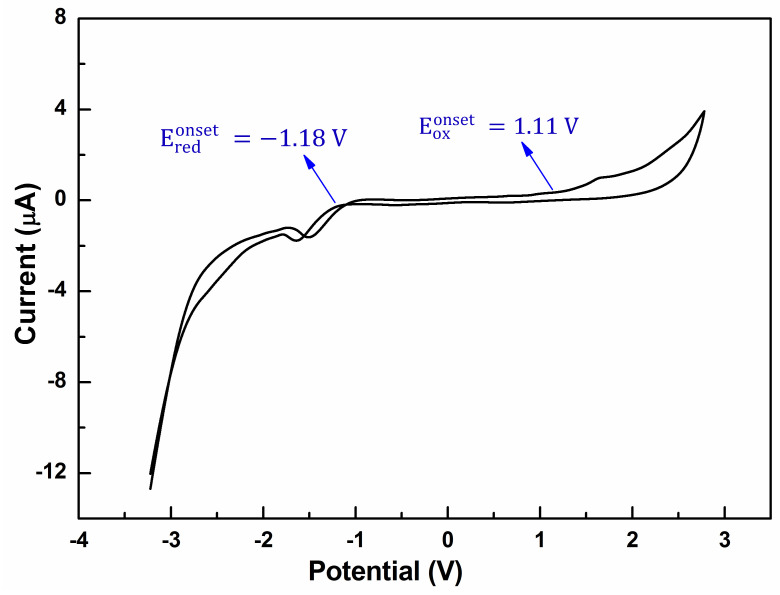
Cyclic voltammogram curve of the oligomer BDT(DBTOTTH)_2_.

To understand the charge transport properties, and to determine the HOMO and lowest unoccupied molecular orbital (LUMO) levels of the oligomer, the redox properties of the oligomer BDT(DBTOTTH)_2_ were investigated by cyclic voltammetry (CV). As shown in Figure 6, the oligomer shows an irreversible oxidation wave, and has an onset oxidation potential of 1.11 eV. It is well known that the HOMO levels of organic compounds can be calculated according to their onset oxidation potentials and the empirical Equation (2).

(2) $$E_{\mathrm{HOMO}}=-e\left(E_{ox}^{onset}+4.4\right)\;\left(\mathrm{eV}\right)$$

Based on Equation (2), the HOMO level of the oligomer BDT(DBTOTTH)_2_ was calculated to be −5.51 eV. The stability of organic semiconducting materials toward oxidative doping is related to their HOMO energy levels. The environmental stability can be improved by lowering the HOMO energy level to minimize the possibility of p-doping by ambient oxygen. Compared with that of pentacene (−4.56 eV) [40], rubrene (−4.69 eV) [41] and sixthiophene (−4.99 eV) [40], the HOMO energy level of the oligomer BDT(DBTOTTH)_2_ is relatively lower. This indicates that the oligomer is oxidatively stable in air. It is a key requirement for organic devices. Furthermore, the HOMO energy level is close to the work function of gold (−5.1 eV) [42]. It suggests that the gold would be the best optimum selection for source and drain electrodes in OFET devices based on the oligomer BDT(DBTOTTH)_2_. The CV curve also includes a reduction wave, an onset reduction potential is at −1.18 V. According to the empirical Equation (3), the determined LUMO energy level of the oligomer is −3.22 eV.

(3) $$E_{\mathrm{LUMO}}=-e\left(E_{red}^{onset}+4.4\right)\;\left(\mathrm{eV}\right)$$

#### 2.3.4. Thermal Analysis

**Figure 7:**
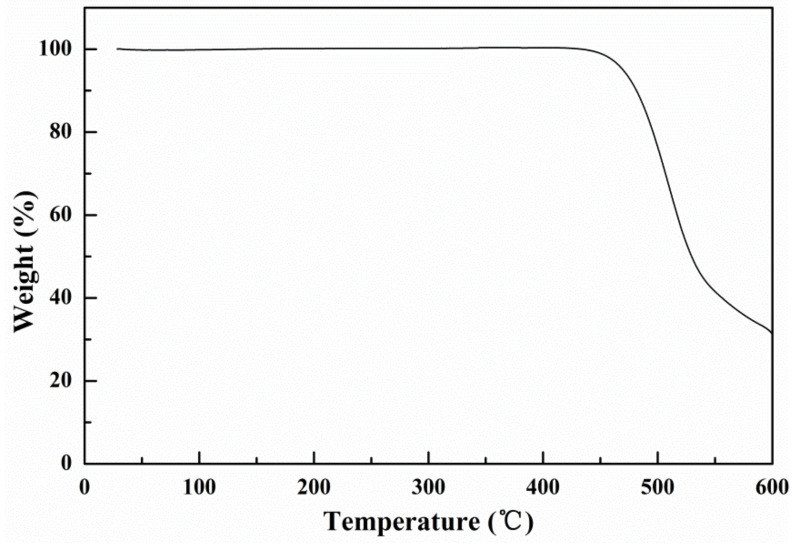
TGA curve of the oligomer BDT(DBTOTTH)_2_.

The thermal stability of the oligomer BDT(DBTOTTH)_2_ was evaluated by TGA in N_2_ atmosphere. As shown in Figure 7, the oligomer exhibited good thermal stability and the losing less than 5% of weight was observed higher than 400 °C. It indicates that the oligomer BDT(DBTOTTH)_2_ exhibits good thermal stability.

#### 2.3.5. OFET Performance

**Figure 8:**
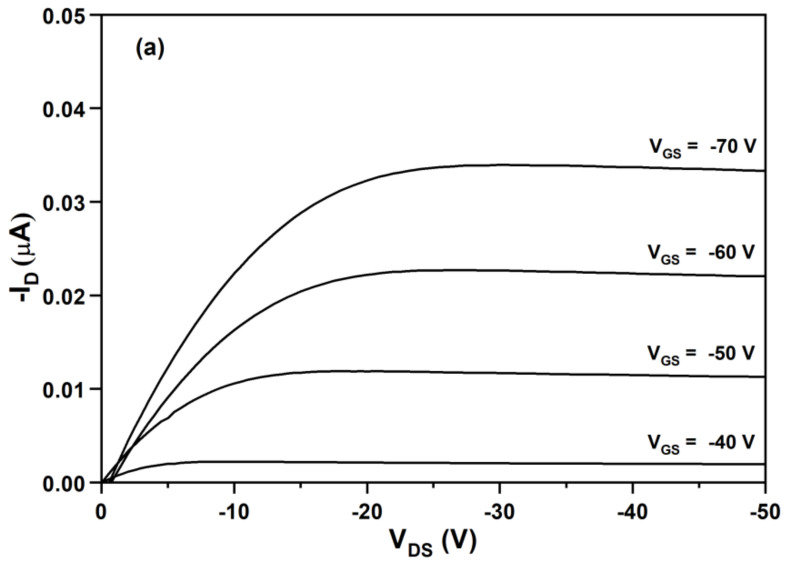

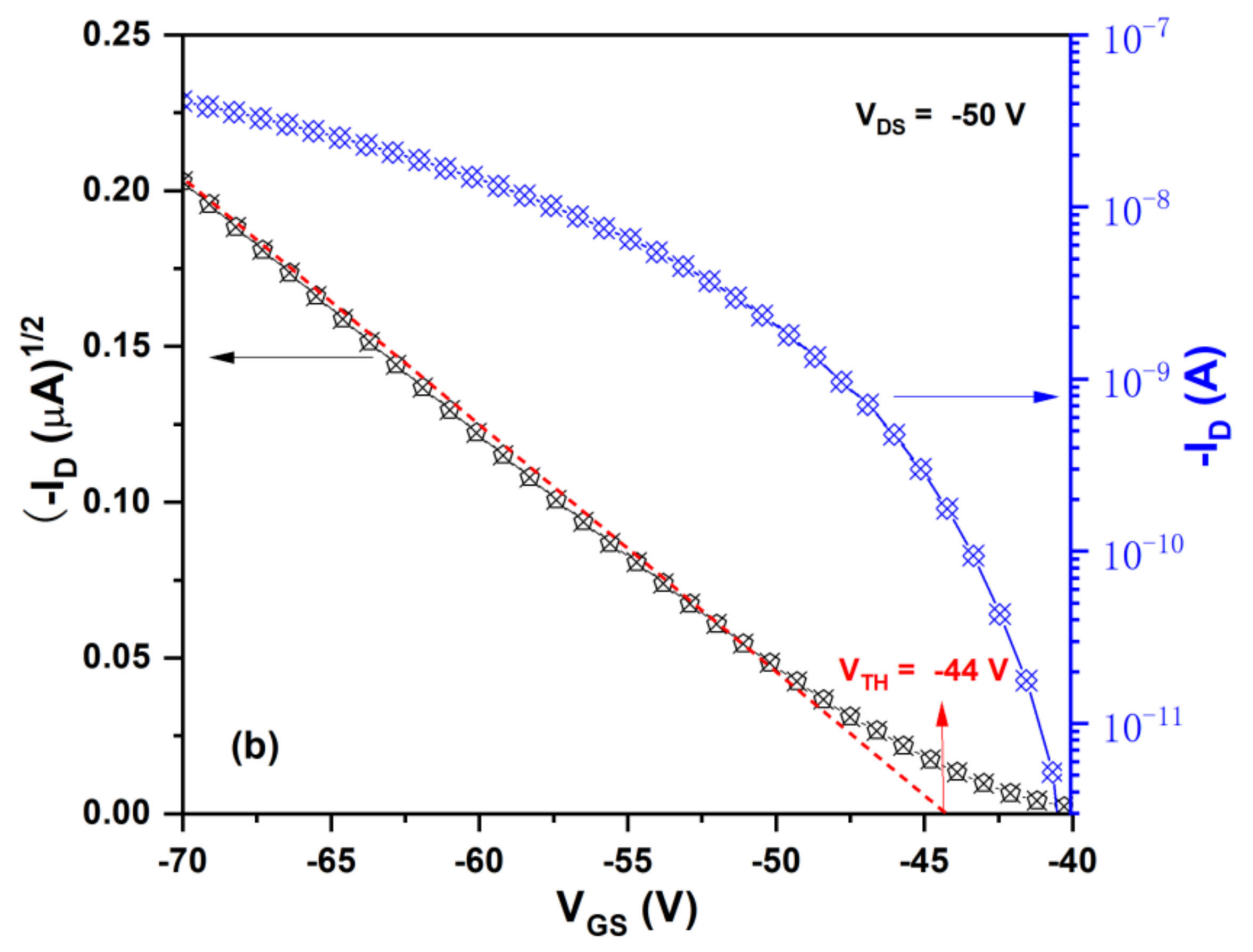
(**a**) Output characteristics and (**b**) transfer characteristics of the OFET device based on BDT(DBTOTTH)_2_.

Figure 8a shows the relationships between the drain-current (I_D_) and drain-source voltage (V_DS_) at different gate-source voltages (V_GS_) from −40 to −70 V for the OFET device using OTS/SiO_2_ as the insulating dielectric layer. The function of the transistor with the negative gate voltage range suggests that BDT(DBTOTTH)_2_ is a p-type semiconductor material. The output characteristics show a good saturation behavior and clear saturation currents that are quadratic to the gate bias. Furthermore, as a more negative V_GS_ was used, more holes were induced in the accumulation layer of the organic semiconductor. As a result, an increased I_DS_ was achieved. The most critical properties of an OFET device are the charge mobility (*μ_sat_*) and I_on_/I_off_ current ratio. The charge mobility is the average drift velocity per unit electric field, and it can be calculated in the saturation regime using following Equation (4),

(4) $$\mu {\;}_{sat}\left(V_{GS}\right)=\frac{2L}{WC_{i}}{\left[\frac{\partial \sqrt{I_{D}\left(V_{GS}\right)}}{\partial V_{GS}}\right]}^{2}\;\;\;\;(\left|V_{DS}\right|\geq \left|V_{GS}-V_{TH}\right|)$$

where *μ_sat_* is the field-effect mobility, W the channel width (500 μm), L the channel length (50 μm), *C_i_* the capacitance of the insulator layer, I_D_ the drain-current, *V_GS_*, *V_DS_* and *V_TH_* are the gate voltage, drain-source voltage and threshold voltage. In order to calculate the field-effect mobility, *V_TH_* were determined firstly. Figure 8b shows the relationship between the square root of I_D_ and V_GS_ at *V_D__S_* = −50 V. From the slope of the plot of (*I_D_*)^1/2^ versus *V_GS_*. The *V_TH_* of the OFET device was determined to be −44 V. Using Equation (4), the calculated mobility value of the OFET device was 1.6 × 10^−3^ cm^2^/Vs. Its I_on_/I_off_ current ratio, which was defined as the ratio of current flow between the source and drain when there was no gate bias and the current flow at maximum gate bias, was higher than 1.0 × 10^4^. The mobility value of the OFET device is really not high. It is well known that the performance of OFET devices depends not on the molecule structure of semiconductor materials, but also on other experimental factors such as purity, dielectric layer, thickness and morphology of semiconductor layer, and electrode and structure of the device. Although the mobility of the OFET device based on the oligomer BDT(DBTOTTH)_2_ is lower than those of many reported thiophe-containing oligomers, the further improvements of its OFET performance can be expected in our future study.

## 3. Conclusions

A novel π-conjugated D-A-D-A-D type thiophene-containing oligomer BDT(DBTOTTH)_2_ comprising a benzo[*1,2-b:4,5-b′*]dithiophene donor core, two DBTSO acceptor intermediaries and two end-capped hexyl dithienyl donor units were designed and synthesized. The oligomer exhibits the energy band gap of 2.25 eV, the HOMO level of −5.51 eV, and LUMO level −3.22 eV. The remarkable shifts in UV-vis and PL spectra for the thin film relative to its corresponding solution indicate the existence of intermolecular π-π stacking in the solid state. The oligomer has good solubility owning to the contribution of hexyl and 2-ethyl-hexyloxy groups, and it can be used to fabricate OFET devices by the spin coating method. The oligomer is p-type semiconductor material, and its OFET device shows the mobility of 1.6 × 10^−3^ cm^2^/Vs.

## Data Availability

Not applicable.

## Sample Availability

Samples of the compounds are not available from the authors.
